# Difference of xenoreactive immune response and patterns of calcification of porcine and bovine tissues in alpha-gal KO and wild type mouse implantation model

**DOI:** 10.1186/1749-8090-8-S1-O126

**Published:** 2013-09-11

**Authors:** J Saeromi, K Min Seok, K Yong Jin, L Cheong, L Hong Gook

**Affiliations:** 1 Department of Thoracic and Cardiovascular Surgery, Seoul National University Hospital, Seoul National University College of Medicine, Seoul, Korea

## Background

Bioprostheses made of bovine and porcine tissue are widely used during surgery in the cardiovascular disease. However, the durability of bioprostheses is limited due to immune reaction-mediated cardiac valve destruction upon post-operative follow-up. The objective of this study is to analyze the tissue failure patterns by difference of species and their structural characteristics.

## Methods

The various xenogeneic tissues (porcine pericardium, aortic valve, aortic wall and bovine pericardium, aortic valve, aortic wall) were fixed in 0.5% glutaraldehyde solution and implanted into the subcutaneous tissue of the wild type-mouse and the KO mouse for 3 months. The implanted mice were harvested and studied for the histopathology, amounts of calcification and immune responses by serum ELISA.

## Results

The titer of xenoreactive antibody (IgG, IgM) after the implantation with xenogeneic tissue in alpha-gal KO mouse tended to increase, with IgM level decreasing faster than IgG level thereafter. The calcification patterns and amounts were similar in both bovine and porcine tissue, but the level of calcification was more severe in KO mouse implantation model than the wild type mouse model. The xenoreactive antigeneicity was lowest in the valve leaflet and the calcification was most severe in aortic wall in both KO mouse & wild type mouse by histologic and quantitative calcium analysis.

## Conclusion

There was different antigenic response among the different xenogenic tissues. The removal of alpha-gal antigenecity is strongly advised and the choice of the xenogeneic tissue with low degree of antigenicity and calcification seems to be important to prevent the degenerative failure of bioprostheses.

